# Species Distribution of *Candida* Isolates in Amniotic Fluid, Vaginal Discharge, and Lochial Cultures in Obstetric and Gynecological Patients: A Retrospective Single-Center Study

**DOI:** 10.3390/pathogens15080797

**Published:** 2026-07-28

**Authors:** Silvia Gabriela Ionescu, Elena Teona Cosovanu, Cristina Daniela Dimitriu, Costin Damian, Diana Costin, Ancuta Goriuc, Elena Porumb Andrese, Demetra Gabriela Socolov, Mihaela Grigore, Luminita Smaranda Iancu, Irina Draga Caruntu, Ramona Gabriela Ursu

**Affiliations:** 1 Grigore T. Popa University of Medicine and Pharmacy Iasi, 700115 Iași, Romania; 2 “Cuza-Vodă” Clinical Hospital of Obstetrics and Gynecology, 700038 Iași, Romania; 3 National Institute of Public Health, Iasi Regional Center for Public Health, 700465 Iași, Romania

**Keywords:** *Candida* spp., amniotic fluid, vaginal discharge, lochia, fungal culture, vulvovaginal candidiasis, *Candida* colonization, species distribution, non-*albicans Candida*

## Abstract

(1) Background: *Candida*-positive cultures in pregnancy and the postpartum period are frequently identified in obstetric and gynecological practice. This study evaluated species distribution and associated maternal and obstetric variables in amniotic fluid, vaginal discharge, and lochial cultures. (2) Methods: A retrospective, single-center, cross-sectional comparative study was conducted at the “Cuza Vodă” Clinical Hospital of Obstetrics and Gynecology, Iași, including *Candida*-positive samples collected in 2024 (242 amniotic fluid, 2020 vaginal discharge, and 827 lochial isolates). Fungal identification was performed using Sabouraud and chromogenic media, with species-level identification in most cases and classification as *Candida* spp. when differentiation was not possible. (3) Results: *Candida albicans* predominated across all compartments (69.8%, 55.3%, and 66.9%, respectively), followed by non-*albicans* species, mainly *Pichia kudriavzevii*. Vaginal discharge cultures, obtained from both pregnant and non-pregnant women and analyzed after stratification by pregnancy status, showed multiple significant associations with maternal and obstetric variables; apparent associations with fetal variables were largely attributable to the inclusion of non-pregnant women and were not retained within the pregnant stratum. Amniotic fluid and lochial cultures demonstrated limited associations. Older maternal age was the only consistent independent correlate of non-*albicans Candida* isolation, whereas the remaining associated variables were compartment-specific. Multivariable models identified compartment-specific correlates of non-*albicans Candida* isolation but showed limited discriminatory performance, with low sensitivity in most strata. (4) Conclusions: *Candida* species distribution is compartment-dependent, with greater clinical heterogeneity in vaginal discharge and more limited patterns in amniotic fluid and lochia. These culture-positive findings support species-level identification for microbiological surveillance and provide perinatal microbiological context for future integrated maternal–neonatal studies; because antifungal susceptibility and neonatal outcomes were not assessed, no therapeutic or neonatal inferences can be drawn.

## 1. Introduction

Fungal pathogens and antifungal resistance are increasingly framed as public-health priorities, and the World Health Organization fungal priority pathogens list explicitly includes multiple medically important yeast taxa, with *Candida albicans* categorized as critical priority and other clinically relevant yeasts (e.g., *Nakaseomyces glabratus*, *Candida tropicalis*, *Candida parapsilosis*, and *Pichia kudriavzevii*) prioritized because of their burden and resistance implications. In parallel clinical practice, candidiasis is understood as disease resulting from overgrowth of endogenous yeasts that normally colonize human sites (including the vagina), with phenotypes spanning localized mucosal infection to invasive disease in vulnerable hosts; pregnancy and hormone-associated physiologic changes are recognized risk enhancers for vaginal candidiasis [1,2].

In obstetrics, *Candida* is clinically relevant not only as a cause of symptomatic vaginitis, but also because rare intrauterine involvement can be associated with severe perinatal outcomes. A focused synthesis of *Candida* chorioamnionitis cases reports prematurity and substantial fetal–neonatal morbidity and mortality, highlighting that intra-amniotic infection, although uncommon, can carry high clinical stakes. At the population level, evidence syntheses of vulvovaginal yeast infection during pregnancy emphasize biologic plausibility via inflammation, but the aggregated epidemiologic signal for adverse outcomes remains uncertain because of heterogeneity, frequent high risk of bias, and limited confounding control in the primary literature [3,4,5]. The pregnancy vaginal environment is also clinically important because species distribution and antifungal susceptibility are not static. More broadly, recent *Candida* literature highlights the importance of accurate molecular detection, recognition of antifungal resistance mechanisms, and implementation of antifungal stewardship programs [6]. In addition, pregnancy-microbiome literature links alterations in vaginal microbial ecology (including loss of protective *Lactobacillus* dominance and increased community diversity) with multiple obstetric complications, reinforcing that compartmental microbiologic states can be clinically informative [7].

Clinical guidance for vulvovaginal candidosis emphasizes disciplined diagnosis (because symptoms are not sufficiently specific), with escalation to culture and species identification in recurrent or complicated presentations; pregnancy is consistently treated as a special population in which topical azole therapy for 7 days is generally preferred. These principles align with sexually transmitted infection guidance frameworks and expert reviews that position yeast culture as a diagnostic reference standard while also describing the expanding—but still scenario-dependent—role of newer molecular assays. Collectively, guideline-based perspectives support laboratory-confirmed, species-informed perinatal *Candida* characterization in settings where maternal–fetal risk assessment matters [8,9,10].

Accurate interpretation of species distribution also requires taxonomic clarity, because several clinically relevant yeasts historically reported within the genus *Candida* have been reclassified (including *Candida krusei* as *Pichia kudriavzevii* and *Candida glabrata* as *Nakaseomyces glabratus*), and inconsistent adoption of updated names can create clinician-facing ambiguity if not handled transparently. Pregnancy-focused reviews further discuss associations between vulvovaginal candidiasis and adverse obstetric events (including PROM, chorioamnionitis, preterm birth, and puerperal infections) and explicitly note that the most frequent pathway to intrauterine infection is ascending genital tract infection, while also emphasizing that evidence remains insufficient to conclude that systemic therapy prevents major outcomes in all contexts. In parallel, management is increasingly complicated by fluconazole-resistant vulvovaginal candidosis, reinforcing the need for targeted regimens and alternative options, alongside randomized trial evidence evaluating topical treatments in pregnancy [11,12,13,14]. Finally, the biological plausibility of “shifting niches” across intrauterine, intrapartum, and postpartum compartments is strengthened by microbiome studies showing that delivery is accompanied by abrupt remodeling of urogenital microbial communities, with substantial postpartum transitions away from *Lactobacillus*-dominant states, and by paired maternal–infant analyses suggesting coordinated maternal vaginal and neonatal microbial shifts in the context of vulvovaginal candidiasis. Contemporary antenatal studies further show that colonization/infection patterns—and susceptibility profiles—vary across settings, while systematic review evidence on *Pichia kudriavzevii* highlights intrinsic fluconazole resistance and the need for timely identification and susceptibility-informed care. In addition, recent amniotic fluid susceptibility data demonstrate that even in intra-amniotic *Candida*, species diversity and resistance can be detected, supporting species-level evaluation in obstetric microbiology [15,16,17,18,19,20].

From a neonatal perspective, microbiological assessment does not begin exclusively after birth. Fetal and neonatal microbial exposure may be shaped by antenatal, intrapartum, and early postpartum compartments, including amniotic fluid, the maternal genital tract, and postpartum genital secretions. Although maternal *Candida* isolation does not necessarily imply neonatal infection, species-level characterization of *Candida*-positive perinatal cultures may provide clinically relevant information regarding potential microbial exposure at the maternal–fetal and maternal–neonatal interfaces. Within this framework, evaluating *Candida* species distribution in amniotic fluid, vaginal discharge, and lochial cultures may contribute to a broader understanding of perinatal fungal ecology and may support future studies integrating maternal and neonatal microbiological data.

Aim: This retrospective study evaluated the species distribution of *Candida* isolates in amniotic fluid, vaginal discharge, and lochial cultures collected over a one-year period, and explored associations between species grouping (*Candida albicans* versus non-*albicans Candida*) and maternal and obstetric characteristics. Within each sample type, we further examined which characteristics were independently associated with non-*albicans Candida* isolation. By characterizing *Candida*-positive compartments that may contribute to fetal or neonatal microbial exposure, the study also aimed to provide a perinatal microbiological framework that may inform future integrated maternal–neonatal investigations.

## 2. Materials and Methods

### 2.1. Study Design and Setting

A retrospective, single-center, cross-sectional comparative study was conducted at the “Cuza Vodă” Clinical Hospital of Obstetrics and Gynecology, Iași, Romania. All samples were collected over a one-year period (January–December 2024) as part of routine microbiological investigations. The unit of analysis was the individual *Candida*-positive culture (isolate); amniotic fluid, vaginal discharge, and lochial specimens were treated as three independent sample types and analyzed separately, rather than as paired or longitudinally collected samples obtained from the same women across the perinatal period. Because the isolates were retrieved from routine laboratory records, it could not be reliably determined whether an individual patient had contributed more than one positive culture, either within or across sample types; consequently, repeated samples from the same patient may be present in the dataset, and the results should be interpreted at the level of the isolate rather than the individual woman.

### 2.2. Sample Collection and Microbiological Analysis

**Figure 1:**
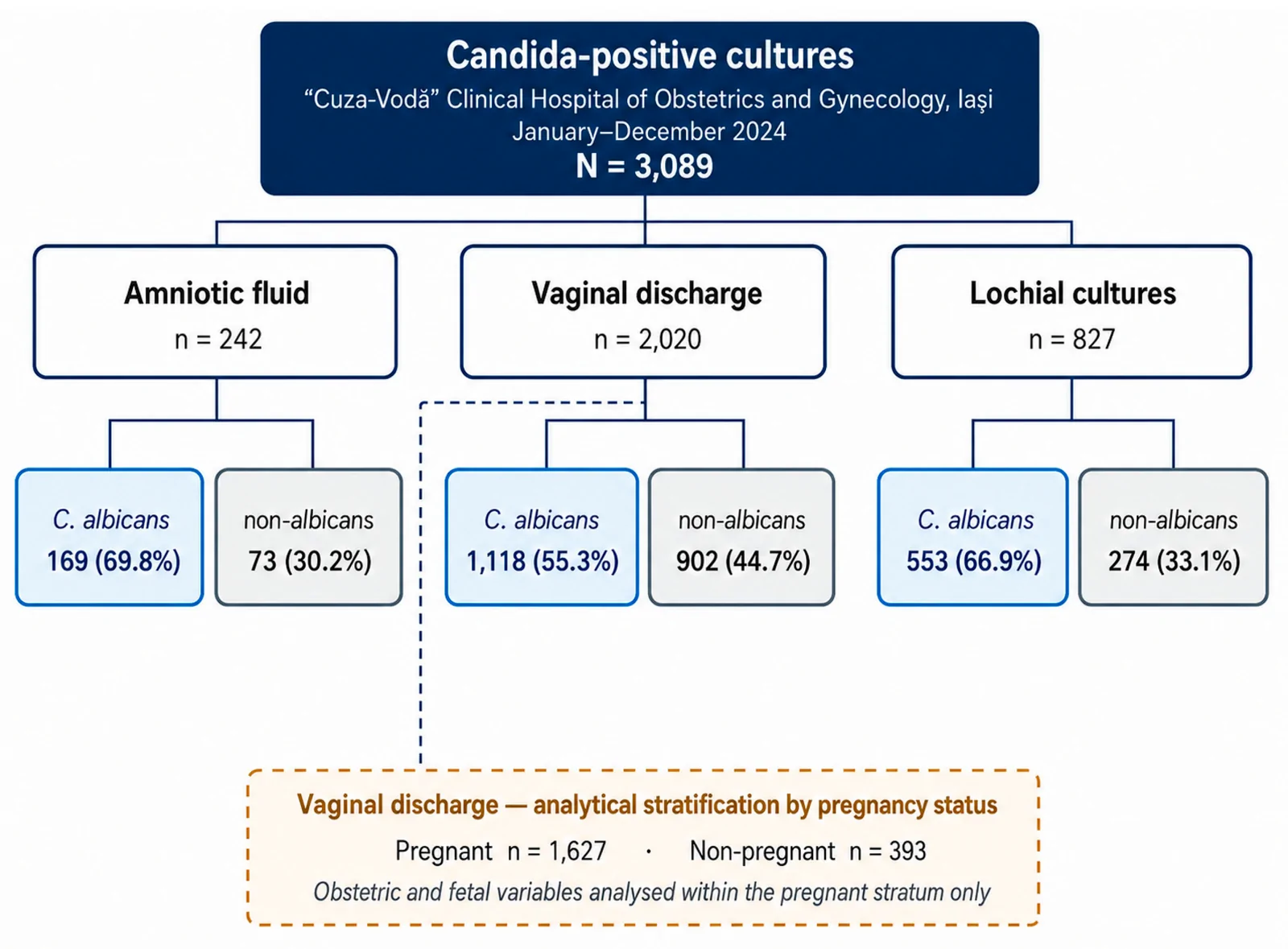
Study population and analytical workflow. A total of 3089 *Candida*-positive cultures collected between January and December 2024 were included and analyzed according to sample type: amniotic fluid, vaginal discharge, and lochial cultures. Vaginal discharge cultures were further stratified by pregnancy status; obstetric and fetal variables were analyzed only within the pregnant stratum.

A total of 242 amniotic fluid, 2020 vaginal discharge, and 827 lochial *Candida*-positive cultures were included (Figure 1). Cultures were requested during routine clinician-directed care. Because the retrospective records did not consistently document the clinical indication for culture or relevant exposures, including recent antibiotic or antifungal therapy, hospitalization, cervical cerclage, and immunosuppression, these factors could not be reliably reconstructed or included in the analyses. Specimens were inoculated onto Sabouraud Dextrose Agar (Oxoid Ltd., Basingstoke, UK) for primary fungal isolation and onto CHROMagar™ *Candida* (CHROMagar, Paris, France), on which presumptive species differentiation was based on colony colour and morphology (for example, green colonies for *Candida albicans* and pink, rough colonies for *Pichia kudriavzevii*). Definitive species-level identification was performed using the VITEK^®^ 2 Compact 60 automated system with VITEK^®^ 2 YST identification cards (REF 21343), operated with VITEK^®^ 2 software version 9.04.4 (bioMérieux, Marcy-l’Étoile, France), according to the manufacturer’s instructions. All *Candida*-positive isolates were processed through the same identification pathway, comprising primary isolation, presumptive differentiation on chromogenic medium, and confirmatory VITEK^®^ testing; a species-level designation was recorded only when the VITEK^®^ system returned a definitive species-level identification. Chromogenic morphology served only as a presumptive step, and the VITEK^®^ result was treated as definitive. When the two were discordant, the species assignment followed the VITEK^®^ identification. Isolates for which VITEK^®^ 2 did not provide a definitive species-level identification were reported as *Candida* spp., accounting for 8.7% of amniotic fluid isolates, 14.0% of vaginal discharge isolates, and 9.7% of lochial isolates. The retrospective database retained the final laboratory identification but not individual VITEK^®^ confidence scores, low-discrimination outputs, or systematic records of CHROMagar™–VITEK^®^ discordance; therefore, these parameters could not be quantified. MALDI-TOF mass spectrometry and sequencing were not used during the study period. Named rare species, including *Candida parapsilosis*, were reported separately, and reference *Candida* strains were processed in parallel for quality control. Because these specimens were retrieved from routine laboratory records that captured only *Candida*-positive results, the total number of amniotic fluid, vaginal discharge, and lochial cultures performed during the study period could not be ascertained. Accordingly, this analysis characterizes the species distribution among *Candida*-positive cultures only and does not permit estimation of culture positivity, prevalence, or incidence. For the exploratory analyses, the binary outcome contrasted cultures reported exclusively as *Candida albicans* with all other *Candida*-positive cultures. Therefore, cultures containing both *C. albicans* and a confirmed non-*albicans Candida* species (mixed cultures; *n* = 6: 1 amniotic fluid, 2 lochial, and 3 vaginal discharge cultures) were assigned to the non-*albicans* analytical category because they could not be considered exclusively *C. albicans*. For species-level tabulation, each mixed culture was counted under its identified non-*albicans* component. These cultures were counted in the non-*albicans* group because the presence of any non-*albicans* component, several of which show reduced azole susceptibility, is the finding of greatest therapeutic relevance. The effect of this decision was tested directly in a sensitivity analysis.

### 2.3. Study Variables

Demographic, clinical, obstetric, and fetal data were collected from medical records, including maternal age, place of residence, body mass index (BMI), smoking status, obstetric history (gravidity, parity, previous caesarean section, infertility), pregnancy characteristics (gestational age, membrane status, onset of labor, complications), maternal comorbidities (diabetes mellitus, hypertensive disorders, thyroid disorders, inherited thrombophilia or thrombocytopenia, renal disease, hepatitis B or C, HIV infection, ocular disorders, uterine leiomyoma, history of neoplasia, intervertebral disc herniation, hepatic abscess, cholelithiasis, sinus tachycardia, bronchial asthma, and gestational edema), and fetal outcomes. Isolates were classified as *Candida albicans* or non-*albicans Candida*. Body mass index (BMI) was based on the weight and height recorded at the time of sampling and categorized according to WHO criteria. For pregnant and postpartum women, overweight and obesity reflected pre-existing weight-status categories documented in the medical records and were not based on gestational or early postpartum weight gain; for non-pregnant women, BMI was calculated from the weight and height recorded at the time of sampling. For exploratory comparative and multivariable analyses, cultures were classified as exclusively *Candida albicans* or as an aggregated non-*albicans Candida* category comprising named non-*albicans* species, *Candida* spp. isolates without definitive species-level identification, and mixed cultures containing both *C. albicans* and a non-*albicans* species. Species-level distributions were summarized separately, and species-specific adjusted models were not fitted because several taxa were too infrequent to yield stable estimates; therefore, this binary grouping should not be interpreted as implying biological equivalence among taxa. In the vaginal discharge group, cultures were obtained from both pregnant women (obstetric setting) and non-pregnant women (gynecological setting) across the full age range, including peri- and post-menopausal patients; this group was therefore treated as a mixed gynecological–obstetric population and was stratified a priori by pregnancy status into pregnant and non-pregnant subgroups. Obstetric and fetal variables (gestational age, status of fetal membranes, onset of labor, fetal presentation, fetal conditions, and fetal outcome) were defined only for pregnant women; for non-pregnant women these variables were treated as not applicable and were excluded from the corresponding comparisons rather than encoded as a distinct category.

### 2.4. Statistical Analysis

Statistical analysis was performed using IBM SPSS Statistics version 26.0 (IBM Corp., Armonk, NY, USA). Categorical variables were expressed as frequencies and percentages. Comparisons between *Candida albicans* and non-*albicans Candida* groups were performed using the Chi-square test or Fisher’s exact test, as appropriate. To identify independent predictors of non-*albicans Candida* isolation, multivariable logistic regression models were constructed using an a priori variable-entry strategy rather than univariate selection alone. Clinically continuous variables (maternal age, gestational age, and parity) were entered as continuous predictors a priori, irrespective of their categorical univariate result, because the categorical groupings used for descriptive tabulation were not intended to govern the functional form of these variables in the regression models; univariate screening (retention of variables with *p* < 0.05) was therefore applied only to categorical candidate predictors. Multicollinearity among the continuous predictors, in particular maternal age and parity, which are plausibly correlated, was assessed using the variance inflation factor (VIF) and tolerance; across the four multivariable models the maximum VIF was 1.30 and the minimum tolerance 0.77, consistent with the absence of problematic collinearity. Results were reported as odds ratios (OR) with 95% confidence intervals (CI). Model performance was evaluated using classification accuracy, sensitivity, specificity, Nagelkerke R^2^, and the area under the receiver operating characteristic curve (AUC). Statistical significance was defined as *p* < 0.05. As a sensitivity analysis, the association between maternal age, the principal correlate identified across the primary models, and non-*albicans* isolation was re-examined in a univariable logistic model after excluding the six polymicrobial co-isolations and all genus-level isolates lacking a species-level identification. For the vaginal discharge cultures, all associations were assessed separately within the pregnant and non-pregnant strata, and obstetric and fetal variables were analyzed exclusively within the pregnant stratum. Categories corresponding to women who were not currently pregnant were treated as not applicable and were excluded from obstetric and fetal comparisons, whereas observations recorded as not recorded were handled as missing data. Separate multivariable logistic regression models were fitted for the pregnant and non-pregnant strata, with obstetric and fetal predictors entered only in the pregnant-stratum model. In all models the outcome was coded as non-*albicans Candida* (=1) versus *Candida albicans* (=0). To limit overfitting and avoid unstable estimates from sparsely populated categories, maternal age, gestational age, and parity were entered as continuous variables, and only biologically plausible, sufficiently populated predictors were retained; binary predictors were referenced to their absent or normal category (rural residence and absence of the comorbidity). Maternal BMI, for which approximately 98% of cultures originated from normal-weight women, and other extremely rare categories were not entered into the multivariable models.

Throughout the analysis, true clinical categories, not-applicable observations, and missing values were treated as distinct. Missing data were not imputed, and each variable was analysed on a complete-case basis. For antepartum pregnancy complications and fetal conditions, the absence of a documented finding was recorded as a true negative (“None”) rather than as missing data.

The chi-square test was applied when all expected cell counts were ≥5. Fisher’s exact test was used for 2 × 2 contingency tables with expected cell counts < 5, whereas a Monte Carlo approximation of the Fisher–Freeman–Halton exact test was used for larger contingency tables with expected cell counts < 5. Reported *p*-values are two-sided and unadjusted. Categorical variables with sparse or clinically redundant categories were collapsed into clinically meaningful groups before testing; for example, antepartum complications and fetal conditions were reduced to their principal categories, with the remaining rare events combined. All association analyses were exploratory and hypothesis-generating. To account for the large number of comparisons performed across the three sample types, the Benjamini–Hochberg procedure was applied to control the false discovery rate at 5% within each sample type, and associations are interpreted primarily in the light of these false-discovery-rate-adjusted results. Owing to the number of tests performed and the limited size of the amniotic fluid and lochial cohorts, associations with marginal *p*-values (approximately 0.01–0.05) that did not survive false-discovery-rate correction were interpreted conservatively, as preliminary signals to be confirmed in future studies rather than as established effects.

## 3. Results

### 3.1. Determinants of Candida Species Distribution in Amniotic Fluid

A total of 242 amnioculture isolates were analyzed. *Candida albicans* was the predominant species, accounting for 69.8% of isolates. Among non-*albicans Candida*, the most frequently identified species was *Pichia kudriavzevii* (19.0%), followed by *Candida* spp. (8.7%) and *Nakaseomyces glabratus* (2.5%). In this cohort, species distribution was not associated with most maternal, obstetric, or fetal characteristics, including maternal age, residence, reproductive history, delivery-related variables, and the majority of comorbidities (all *p* > 0.05). In univariate analysis, gestational age (*p* = 0.001), maternal BMI (*p* = 0.029), month of sampling (*p* = 0.040), and inherited thrombophilia (*p* = 0.048) were associated with species distribution; however, the maternal–BMI and thrombophilia associations rested on very few exposed cultures (5 with obesity and 12 with inherited thrombophilia) and, together with month of sampling, did not remain significant after correction for multiple comparisons. Fetal presentation, fetal conditions, and fetal outcome were not associated with species distribution (Table 1). To identify independent predictors, a multivariable logistic regression model was fitted with maternal age, gestational age, and parity entered as continuous variables; maternal BMI and inherited thrombophilia were not modelled because of extremely sparse categories (only 5 isolates originated from women with obesity and 12 from women with inherited thrombophilia), which precludes stable estimation. Older maternal age (OR = 1.07 per year, 95% CI 1.02–1.12, *p* = 0.003) and lower parity (OR = 0.68 per additional birth, 95% CI 0.52–0.90, *p* = 0.007) were independently associated with non-*albicans Candida*, whereas gestational age was not (OR = 0.96 per week, 95% CI 0.91–1.01, *p* = 0.121). The model was statistically significant overall (omnibus *p* < 0.001) but had limited explanatory power (Nagelkerke R^2^ = 0.10); despite an overall classification accuracy of 71.9%, sensitivity for non-*albicans Candida* was low (12.3%, with 97.6% specificity for *Candida albicans*; AUC = 0.65). Consistent with the pre-specified analysis plan, maternal age, gestational age, and parity were entered as continuous predictors a priori; although the categorical maternal–age (*p* = 0.133) and parity (*p* = 0.096) groupings were not significant in the univariate tabulation, the corresponding continuous predictors were independently associated with non-*albicans Candida* in the multivariable model, consistent with graded rather than threshold effects. Collinearity between the continuous predictors was minimal (maternal age VIF = 1.24, parity VIF = 1.30, gestational age VIF = 1.05; all tolerance ≥ 0.77).

### 3.2. Determinants of Candida Species Distribution in Lochial Cultures

A total of 827 lochial culture isolates were included in the analysis. *Candida albicans* was the predominant species, accounting for 66.9% of isolates. Among non-*albicans Candida*, the most frequently identified species was *Pichia kudriavzevii* (21.3%), followed by *Candida* spp. (9.7%) and *Nakaseomyces glabratus* (2.1%). Overall, *Candida* species distribution in lochial cultures showed limited associations with maternal, obstetric, and fetal variables. In univariate analysis, significant associations were observed for maternal age group (*p* = 0.009), place of residence (*p* = 0.035), maternal BMI (*p* = 0.044), and parity group (*p* < 0.001); after Benjamini–Hochberg correction within the lochial cohort, only parity group remained significant, whereas the associations with maternal age group, place of residence, and maternal BMI did not survive correction and are therefore interpreted as exploratory, unadjusted findings. Specifically, non-*albicans Candida* was more frequently identified in older women and among nulliparous and low-parity women, while *Candida albicans* predominated in younger and multiparous patients. Rural residence was more commonly associated with *Candida albicans*, whereas non-*albicans Candida* showed a relatively higher proportion in urban patients. None of the remaining maternal, obstetric, fetal, or comorbidity variables was significantly associated with species distribution (all *p* > 0.05); these included month of sampling, gravidity, previous caesarean section, gestational age, membrane status, onset of labour, antepartum complications, the recorded maternal comorbidities, and fetal presentation, conditions, and outcome (Table 2). In lochial cultures, a multivariable logistic regression model including maternal age and parity was statistically significant (omnibus *p* < 0.001) but had limited explanatory power (Nagelkerke R^2^ = 0.045) and limited discriminatory performance (classification accuracy 65.8%), with low sensitivity for non-*albicans Candida* (3.3%) and high specificity for *Candida albicans* (96.7%), with an AUC of 0.63. Older maternal age was independently associated with higher odds of non-*albicans Candida* (OR = 1.05 per year, 95% CI 1.03–1.08, *p* < 0.001), and lower parity was likewise independently associated with non-*albicans Candida* (OR = 0.76 per additional birth, 95% CI 0.65–0.88, *p* < 0.001), reflecting a higher proportion of non-*albicans* isolates in nulliparous and low-parity women. Place of residence was not retained as an independent predictor. Overall, increasing maternal age and decreasing parity were modest but independent correlates of non-*albicans Candida* in lochial cultures, although the overall predictive performance of the model remained limited.

### 3.3. Determinants of Candida Species Distribution in Vaginal Discharge

**Figure 2:**
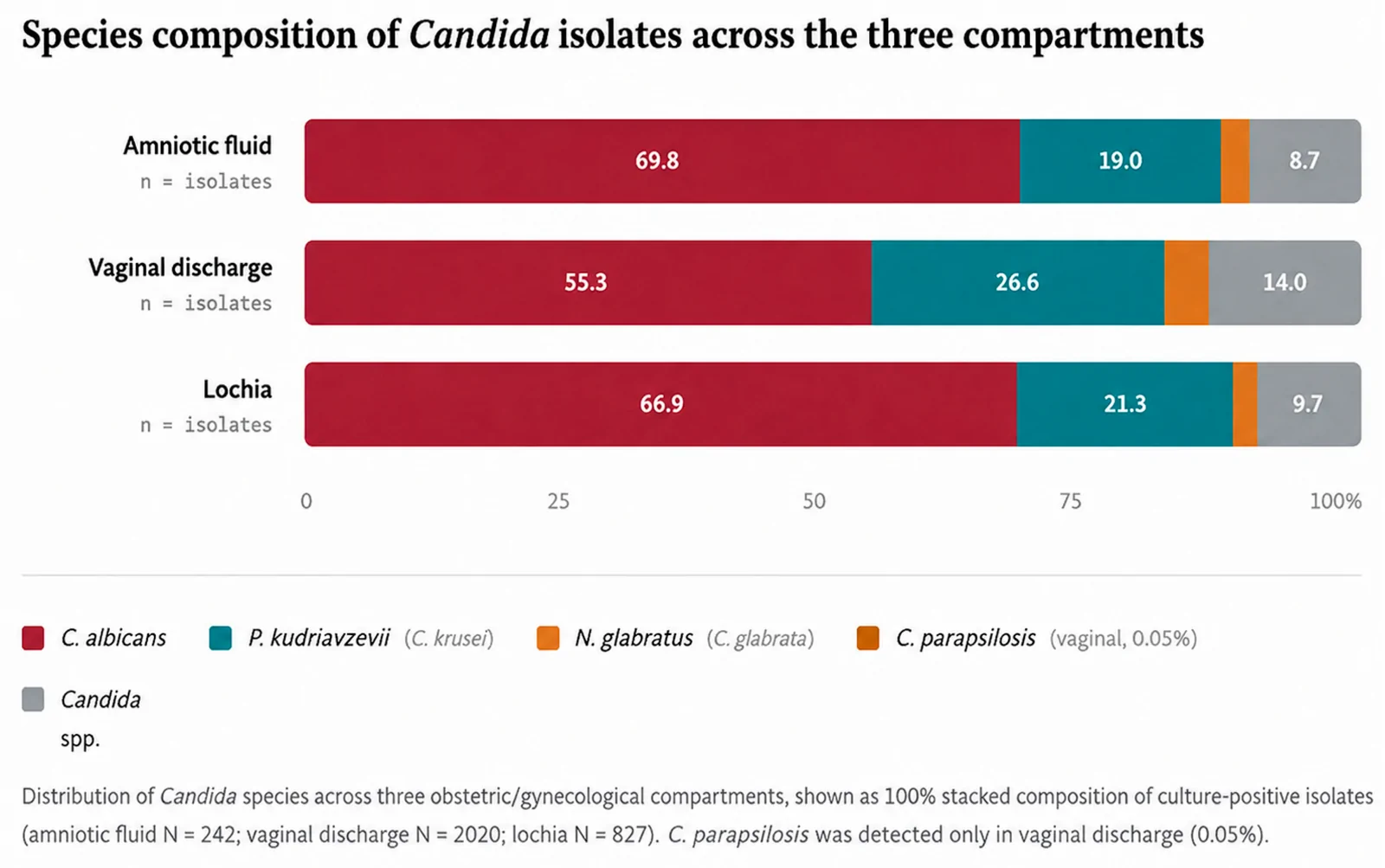
*Candida* species distribution across amniotic fluid, vaginal discharge, and lochial cultures. The figure shows the proportional distribution of *Candida* species among culture-positive isolates within each sample type: amniotic fluid (N = 242), vaginal discharge (N = 2020), and lochial cultures (N = 827). *Candida albicans* predominated in all three sample types, whereas *Pichia kudriavzevii* was the leading non-*albicans Candida* species. *Candida* spp. denotes isolates confirmed at the genus level but not differentiated to species level. *Candida parapsilosis* was detected only in vaginal discharge cultures (0.05%). Polymicrobial co-isolations were counted under their non-*albicans* component, as described in Section 2.

A total of 2020 vaginal discharge isolates were included in the analysis. *Candida albicans* was the predominant species, accounting for 55.3% of isolates. Among non-*albicans Candida*, the most frequently identified species was *Pichia kudriavzevii* (26.58%), followed by *Candida* spp. (13.96%) and *Nakaseomyces glabratus* (4.06%), while *Candida parapsilosis* was rarely detected (0.05%). The species composition across the three sample types is summarized in Figure 2. The distribution of *Candida* species varied significantly according to maternal and obstetric characteristics; because this cohort comprised both pregnant and non-pregnant women, obstetric and fetal variables were assessed only within the pregnant stratum. Maternal age was strongly associated with species distribution (*p* < 0.001), with *C. albicans* predominating in women of reproductive age, particularly between 23–32 years, whereas non-*albicans* species were relatively more frequent in older age groups. In contrast, place of residence and maternal BMI were not significantly associated (*p* = 0.172 and *p* = 0.456), and chronic smoking showed only a borderline, non-significant association (*p* = 0.050). Obstetric history demonstrated significant associations with species distribution. Non-*albicans Candida* was more frequent among nulligravida and nulliparous women, while *C. albicans* predominated in multiparous groups (both *p* < 0.001). Previous caesarean section was also associated with species distribution (*p* = 0.014), whereas in vitro fertilization showed no significant effect (*p* = 0.799). Infertility was significantly associated with a higher proportion of non-*albicans* species (*p* = 0.002). Among current-pregnancy characteristics, assessed within the pregnant stratum, gestational age (*p* = 0.003) and onset of labour (*p* = 0.005) were associated with species distribution, whereas status of the fetal membranes (*p* = 0.858) and antepartum pregnancy complications (*p* = 0.129) were not associated with species distribution. Among maternal comorbidities, significant associations were observed for diabetes (*p* = 0.014), hypothyroidism (*p* = 0.011), and uterine leiomyoma (*p* < 0.001), the latter showing a clear predominance of non-*albicans* species. Other comorbidities, including hypertension, thrombophilia, renal disease, and viral infections, were not significantly associated. In addition, month of sampling (*p* < 0.001) and obstetric hemorrhage (*p* = 0.011) reached statistical significance in univariate analysis; the temporal association most plausibly reflects seasonal and case-mix variation across the study year rather than a biological effect, and the obstetric-hemorrhage association rests on few events, so both are regarded as exploratory. When obstetric and fetal variables were restricted to pregnant women (excluding the not-currently-pregnant cultures), the apparent fetal associations were largely attenuated: within the pregnant stratum, fetal outcome (*p* = 0.297), fetal conditions (*p* = 0.729), and status of fetal membranes (*p* = 0.858) were no longer statistically significant, fetal presentation showed only a borderline, non-significant association (*p* = 0.059), and gestational age remained significant but with a negligible effect size. This indicates that the strong fetal and obstetric associations observed in the pooled vaginal discharge analysis were largely driven by the inclusion of non-pregnant women rather than by a genuine relationship with fetal status. Because the vaginal discharge cohort comprised both pregnant and non-pregnant women, separate multivariable models were fitted for each stratum. In the pregnant stratum (n = 1627), older maternal age (OR = 1.05 per year, 95% CI 1.03–1.06, *p* < 0.001) and the presence of a thyroid disorder (OR = 2.90, 95% CI 1.14–7.35, *p* = 0.025) were independently associated with non-*albicans Candida*, whereas gestational age (OR = 0.99 per week, *p* = 0.062) and parity (OR = 0.93 per birth, *p* = 0.082) showed only non-significant trends toward higher non-*albicans* proportions at earlier gestation and lower parity; the model was significant overall (omnibus *p* < 0.001) but explained little variance (Nagelkerke R^2^ = 0.04), with an accuracy of 61.3%, a sensitivity of 11.6% for non-*albicans Candida*, and a specificity of 92.7% for *Candida albicans* (AUC = 0.60). In the non-pregnant stratum (n = 393), older maternal age remained the only independent predictor (OR = 1.04 per year, 95% CI 1.02–1.06, *p* < 0.001; Nagelkerke R^2^ = 0.10, AUC = 0.68), and non-*albicans Candida* accounted for the majority of isolates in this older subgroup. In this stratum, in which non-*albicans Candida* formed the majority class, the model showed high sensitivity (96.7%) but low specificity (14.2%), the inverse of the sensitivity–specificity pattern observed in the other three models. This sensitivity–specificity inversion should be interpreted as a threshold-dependent consequence of outcome prevalence and class imbalance at the 0.5 probability threshold, rather than as evidence of strong discriminatory performance. Across both strata, increasing maternal age was therefore consistently associated with a higher likelihood of non-*albicans Candida* (Table 3). Excluding the six polymicrobial co-isolations and the genus-level isolates lacking species-level identification did not materially alter the species distribution. In this restricted analysis, older maternal age remained significantly associated with non-*albicans* isolation in lochial (OR 1.04 per year, 95% CI 1.01–1.07) and vaginal discharge cultures (OR 1.06, 95% CI 1.05–1.07), with a concordant direction in amniotic fluid (OR 1.02, 95% CI 0.98–1.07). These findings indicate that the principal association was robust to the classification of co-isolations and genus-level isolates.

The multivariable logistic regression models are summarized in Table 4.

Species-level frequencies of the non-*albicans* isolates in each compartment are detailed in Table 5.

## 4. Discussion

In the present study, a total of 242 amnioculture isolates were analyzed, with *Candida albicans* representing the predominant species (69.8%), followed by non-*albicans Candida*, mainly *Pichia kudriavzevii* and *Nakaseomyces glabratus*. This distribution is consistent with existing literature, which identifies *Candida albicans* as the principal etiological agent in intra-amniotic infections, while non-*albicans* species are less frequently isolated [21,22,23]. Previous reports have emphasized that the detection of *Candida* in amniotic fluid is relatively rare but clinically significant, often associated with preterm labor and adverse perinatal outcomes [21,22]. In our cohort, species distribution showed limited association with most maternal, obstetric, and fetal characteristics; in multivariable analysis, older maternal age and lower parity, rather than gestational age, were the independent predictors of non-*albicans Candida*, and gestational age was not retained once these factors were taken into account. Although intra-amniotic infection has been linked to early gestational complications and preterm labor, our data do not support an independent association between gestational age and species distribution [22,24]. In addition, the intrinsic antifungal activity of amniotic fluid, demonstrated in experimental studies, may partially explain the relatively low diversity observed in our population, as this biological barrier can inhibit fungal proliferation, especially for species such as *Nakaseomyces glabratus* [25]. Case reports of candidal chorioamnionitis further highlight the diagnostic value of amnioculture, which enables accurate species identification even in patients without evident risk factors [23]. In contrast to these reports, our data showed no significant association between *Candida* species and immediate fetal outcomes, consistent with colonization or low-grade infection occurring without overt clinical consequences. Overall, these findings reinforce the predominance of *Candida albicans* among amniotic fluid isolates and indicate that host demographic and clinical characteristics explained little of the variation in species distribution; microbiological evaluation of amniotic fluid nonetheless remains valuable for accurate species identification.

In the lochial sample type, 827 culture isolates were analyzed, again demonstrating the predominance of *Candida albicans* (66.9%), followed by non-*albicans Candida*, mainly *Pichia kudriavzevii* and *Nakaseomyces glabratus*. This pattern is consistent with previous studies indicating that *Candida albicans* remains the dominant species in vaginal and postpartum colonization, despite an overall reduction in yeast isolation rates during the puerperium [26,27]. The absence of significant associations between *Candida* species and most maternal, obstetric, and fetal variables suggests that postpartum colonization is influenced predominantly by local physiological factors rather than systemic maternal characteristics. Indeed, lochial discharge has been shown to exert a mechanical “cleansing” effect, contributing to a transient decrease in vaginal *Candida* colonization during the postpartum period, even in the absence of antifungal treatment [26,27]. Interestingly, although lochial secretions may promote fungal growth under in vitro conditions, the in vivo environment appears to limit colonization, reflecting a complex interaction between biochemical properties and mechanical elimination mechanisms [28].

Within this postpartum context, maternal age and parity emerged as independent predictors of *Candida* species distribution, with non-*albicans Candida* more frequently identified in older and lower-parity (primiparous) women, while *Candida albicans* predominated in younger and multiparous patients. These findings may reflect differences in hormonal milieu, vaginal microbiota stability, and immune adaptation across reproductive stages. However, the overall predictive capacity of the multivariable model remained limited, indicating that these factors exert only a modest influence on species differentiation. The lack of association with gestational variables, delivery-related factors, and maternal comorbidities further supports the hypothesis that postpartum *Candida* dynamics are largely independent of classical obstetric risk factors. Moreover, the absence of significant correlations with fetal outcomes is consistent with the generally benign and transient nature of postpartum *Candida* colonization, as supported by studies demonstrating a physiological decline in yeast isolation rates within 6–8 weeks after delivery [27]. Importantly, this transient reduction does not imply eradication, and recurrence after the resolution of lochia remains possible, highlighting the need for continued clinical awareness beyond the immediate puerperium [26].

In contrast to the relatively stable and physiologically influenced patterns observed in amniotic and lochial samples, the analysis of 2020 vaginal discharge isolates revealed a more complex and clinically dynamic distribution. *Candida albicans* remained the predominant species (55.3%), followed by a substantial proportion of non-*albicans Candida*, particularly *Pichia kudriavzevii*, *Candida* spp., and *Nakaseomyces glabratus*. This distribution aligns with most published data, which consistently identify *C. albicans* as the leading etiological agent of vulvovaginal candidiasis and abnormal vaginal discharge, accounting for approximately 50–70% of isolates in both pregnant and non-pregnant women [29,30,31,32,33,34,35,36,37]. At the same time, the relatively high proportion of non-*albicans* species observed in our cohort supports the growing evidence of a shift toward these species, particularly in pregnancy-associated infections, recurrent disease, and clinically complex cases [38,39,40,41,42,43]. This shift is clinically relevant, as non-*albicans Candida* species are associated with increased virulence factors, biofilm formation, and reduced susceptibility to commonly used antifungal agents, particularly azoles, emphasizing the need for species-level identification [32,38,39,40,41,42,43]. In particular, *Pichia kudriavzevii* (formerly *Candida krusei*), the most frequent non-*albicans* species in the present cohort, is recognized in the literature for intrinsic resistance to fluconazole; because antifungal susceptibility testing was not performed in this study, this consideration derives solely from published data and was not confirmed for the present isolates.

The associations identified between *Candida* species distribution and maternal, reproductive, and gestational characteristics further highlight the multifactorial nature of vaginal *Candida* colonization and species distribution. In our cohort, *C. albicans* predominated in women of reproductive age and in later pregnancy, particularly in the third trimester, consistent with studies demonstrating that hormonal and immunological changes during pregnancy favor fungal overgrowth [29,30,32,35,36]. Conversely, non-*albicans Candida* species were more frequently identified in older women, nulligravida or nulliparous patients, and in earlier gestational stages or non-pregnant states, suggesting a distinct ecological and host-interaction profile [38,39,41,42]. Notably, the vaginal discharge cohort comprised both pregnant and non-pregnant women, the latter frequently of peri- or post-menopausal age, and the apparent associations between *Candida* species and obstetric or fetal characteristics were largely confounded by this case mix: after stratification, fetal outcome, fetal conditions, status of fetal membranes, and fetal presentation were no longer significantly associated with species distribution within the pregnant stratum. The predominance of non-*albicans* species among older and non-pregnant women is consistent with a recognized gynecological pattern of estrogen-dependent shifts in the vaginal mycobiota rather than a perinatal phenomenon. Additional associations with infertility and previous caesarean section further support the role of altered vaginal microbiota and reproductive tract conditions in shaping species distribution. Although maternal comorbidities such as diabetes, hypothyroidism, and uterine leiomyoma showed significant associations in univariate analysis, their lack of independent significance in multivariable models suggests that their overall contribution is modest. Similarly, behavioral and hygiene-related factors, including antibiotic use, sexual activity, and personal hygiene practices, have been reported to influence infection risk, reinforcing the complex interplay between host, microbial, and environmental factors [29,34,37,43,44].

From a clinical perspective, these findings must be interpreted within the broader diagnostic framework of vaginal discharge, which represents a common but nonspecific symptom with a wide differential diagnosis, including both infectious and noninfectious etiologies [45,46]. Importantly, vaginal *Candida* colonization may be asymptomatic, particularly during pregnancy, and does not necessarily indicate clinically significant infection [29,32,42,43]. Nevertheless, when symptomatic, candidiasis is strongly associated with vaginal discharge, pruritus, irritation, and recurrent episodes, significantly affecting quality of life [33,34,35,36,37,43]. In our study, after the analysis was restricted to pregnant women with documented fetal data, *Candida* species were not significantly associated with fetal presentation, fetal conditions, or fetal outcome (all *p* > 0.05); the apparent association of non-*albicans* species with atypical fetal categories in the pooled data reflected the inclusion of non-pregnant women, in whom these variables are undefined, rather than a genuine relationship with fetal status. These fetal analyses are exploratory and do not establish a causal link between *Candida* species and fetal outcome. More broadly, vaginal *Candida* colonization often represents a commensal or low-grade state that may become clinically relevant only under specific conditions. Moreover, rare but severe complications, including ascending infection and chorioamnionitis, have been described even in asymptomatic vaginal candidiasis [44].

Overall, integrating findings across amniotic, lochial, and vaginal samples, our study highlights distinct ecological patterns of *Candida* colonization across the three perinatal sample types examined. While *Candida albicans* consistently remains the dominant species, non-*albicans Candida* are increasingly relevant, particularly in recurrent infections and clinically complex cases. These findings underscore the importance of targeted microbiological evaluation, including culture-based identification, to support accurate diagnosis and species-level surveillance of perinatal *Candida*; because antifungal susceptibility and treatment data were not available, conclusions regarding therapeutic strategies or maternal and neonatal outcomes cannot be drawn [32,35,36,38,39,40,41,43]. After Benjamini–Hochberg correction within each sample type, only gestational age (amniotic fluid) and parity (lochia) retained significance, whereas all vaginal discharge associations that were significant before adjustment also survived correction (12 associations); the remaining marginal associations in the amniotic fluid and lochial cohorts did not survive correction and are therefore best regarded as exploratory. These false-discovery-rate results refer to univariate associations; in multivariable models, older maternal age was the most consistent independent correlate of non-*albicans Candida* across compartments, whereas gestational age was not retained as an independent predictor.

A distinctive feature of the present series is the consistent predominance of *Pichia kudriavzevii* (formerly *Candida krusei*) among non-*albicans Candida* isolates across all three compartments (19.0% in amniotic fluid, 26.58% in vaginal discharge, and 21.3% in lochia), greatly exceeding *Nakaseomyces glabratus* (formerly *Candida glabrata*; 2.5%, 4.06%, and 2.1%, respectively). This distribution diverges from most published vulvovaginal and perinatal series, in which *N. glabratus* is the predominant non-*albicans* species and *P. kudriavzevii* a minor component. In a Lebanese pregnancy cohort, *C. glabrata* accounted for 44.5% of vulvovaginal candidiasis versus 12.1% for *C. krusei* [47]; in a Ghanaian antenatal series, *C. glabrata* was the single most frequently isolated species overall (57.4%) [48]; and in a Vietnamese cohort of non-pregnant women of reproductive age, *C. glabrata* (11.37%) again exceeded *C. krusei* (3.92%) [49]. Because presumptive species differentiation in the present study was initially based on chromogenic colony morphology, a *krusei*-predominant distribution of this magnitude warrants cautious interpretation and cannot be entirely dissociated from the possibility of a species-identification artefact; this consideration is mitigated, although not wholly eliminated, by the confirmatory VITEK^®^ step described in Section 2.2. Several explanations may account for this departure from the *C. albicans* and *N. glabratus* patterns reported in most obstetric and vulvovaginal series. Regional and population-level differences in *Candida* epidemiology are well recognised, and prior azole exposure can favour intrinsically fluconazole-resistant species such as *Pichia kudriavzevii*. Reliance on chromogenic morphology for the initial differentiation, even with subsequent VITEK^®^ confirmation, may also leave some residual species-level misclassification, which again points to molecular identification as the appropriate means of verification. The atypical nature of this finding is further underscored by the WHO fungal priority pathogens list [1], in which *C. albicans* is ranked as critical priority; *N. glabratus*, *C. tropicalis*, and *C. parapsilosis* as high priority; and *P. kudriavzevii* as medium priority. The predominance of a medium-priority yeast over higher-priority non-*albicans* species is unexpected and warrants confirmation in future studies, ideally incorporating molecular identification and antifungal susceptibility testing.

A more systematic reading of the literature nonetheless shows that a *krusei*-predominant pattern, although uncommon, is not without precedent. Among symptomatic pregnant women in Northwest Ethiopia, *C. krusei* (now *P. kudriavzevii*) was the most frequent non-*albicans* isolate at 21.9%, exceeding *C. glabrata* at 17.7% [50]. A similar ranking was reported in pregnant women from Hajjah, Yemen, where *C. krusei* reached 13.58% against 9.87% for *C. glabrata* [51], and in a broader Ethiopian vulvovaginal series in which *C. krusei* (17.2%) clearly outnumbered *C. glabrata* (3.46%) [52]. In these cohorts, as in ours, *P. kudriavzevii* was the leading non-*albicans* species rather than a minor component, at proportions broadly similar to the 19.0–26.6% seen across our three compartments. This convergence with several African and Middle Eastern reports suggests that the present distribution, although atypical for European populations, is epidemiologically plausible and may reflect genuine geographic variation in non-*albicans* ecology, prior azole exposure, or differences in the identification media used. It does not remove the need for molecular confirmation, since most of these comparators, like the present study, relied on chromogenic or phenotypic identification rather than sequencing or MALDI-TOF MS. The predominance of a medium-priority yeast over higher-priority non-*albicans* species is unexpected and warrants confirmation in future studies, ideally incorporating molecular identification, such as ITS or D1/D2 sequencing, and antifungal susceptibility testing.

These findings may also reflect the mixed obstetric–gynecological case-mix and the inclusion of clinician-requested *Candida*-positive cultures. Because clinical indications and recent antimicrobial exposure were unavailable, selection bias and residual confounding cannot be excluded.

### Relevance to Integrated Perinatal and Neonatal Microbiological Assessment

The present study may also be interpreted within the broader framework of integrated perinatal and neonatal microbiological assessment. Within neonatal medicine, this framework is grounded in the systematic microbiological assessment of the newborn, in which pathogen identification, infection surveillance, and risk stratification inform the evaluation of neonatal infectious disease and support the integration of maternal and perinatal microbiological data into neonatal risk assessment [53]. Although neonatal cultures, neonatal colonization, and clinically confirmed neonatal infections were not directly evaluated, the analyzed compartments—amniotic fluid, vaginal discharge, and lochia—represent biologically relevant interfaces through which fetal or neonatal exposure to fungal organisms may occur. Therefore, these findings should be viewed not as direct evidence of neonatal infection, but as microbiological characterization of maternal and perinatal sources that may be relevant for future neonatal risk stratification. Because the present analysis was retrospective and based on routinely collected, culture-positive isolates rather than paired or longitudinally sampled mother–infant dyads, it characterizes species distribution at the level of the isolate and cannot address temporal sequence, within-patient dynamics, or vertical transmission.

Amniotic fluid cultures are particularly important in this framework because they represent the compartment most directly related to the intrauterine environment. The predominance of *Candida albicans* in amniotic fluid isolates confirms its major role among *Candida*-positive intra-amniotic cultures, while the identification of non-*albicans Candida*, especially *Pichia kudriavzevii*, highlights the need for species-level reporting. In the context of neonatal microbiological evaluation, such information may be useful because fetal exposure to different *Candida* species may have distinct clinical implications, particularly in preterm or otherwise vulnerable neonates. However, in the absence of paired neonatal cultures, the present data cannot establish vertical transmission or neonatal disease.

Vaginal discharge cultures provide complementary information regarding maternal genital tract colonization. During birth, the newborn may be exposed to maternal vaginal microorganisms, and *Candida*-positive vaginal cultures may therefore represent a relevant component of the perinatal microbial environment. In the present study, *Candida albicans* remained the predominant species, but the substantial proportion of non-*albicans Candida* suggests that species-level identification may be clinically informative, particularly in complicated, recurrent, symptomatic, or high-risk obstetric settings. This is relevant to future neonatal microbiological assessment because maternal genital tract colonization may be considered alongside neonatal cultures, delivery mode, gestational age, rupture of membranes, intrapartum antimicrobial exposure, and neonatal clinical status.

Lochial cultures may reflect the early postpartum genital microbial environment. Although the relationship between lochial *Candida* isolation and neonatal infection is indirect and currently uncertain, postpartum maternal colonization may contribute to the microbial context in which early neonatal exposure occurs. From the perspective of complex neonatal microbiological evaluation, lochial cultures may therefore be considered as part of the broader maternal–neonatal microbial interface, particularly in studies designed to evaluate early-life colonization, environmental exposure, or postpartum infectious risk.

Overall, the findings support the concept that neonatal microbiological assessment should be interpreted within a wider perinatal context. The study does not demonstrate neonatal candidiasis, neonatal sepsis, or vertical transmission; however, it characterizes *Candida* species in maternal and perinatal compartments that may influence fetal or neonatal microbial exposure. Future research should integrate paired maternal and neonatal sampling, neonatal culture or molecular testing, antifungal susceptibility profiling, and detailed clinical outcome data in order to determine whether specific maternal *Candida* species, specific perinatal compartments, or non-*albicans Candida* profiles are associated with neonatal colonization, early-onset fungal infection, prematurity-related morbidity, or other neonatal outcomes.

Several methodological limitations should be acknowledged when interpreting these findings. Because the amniotic fluid, vaginal discharge, and lochial isolates were obtained from independent routine cultures rather than from paired or longitudinally followed women, the present analysis does not capture within-patient microbiological transitions across the perinatal period and should be read as a cross-sectional comparison of species distribution between three sample types. In addition, because repeated positive cultures from the same patient could not be reliably identified and removed from the laboratory dataset, an individual woman may have contributed more than one isolate; the unit of analysis is therefore the isolate rather than the patient, and potential clustering of isolates at the patient level could not be accounted for in the statistical models. Because this within-patient clustering could not be modelled, the assumption of independent observations that underlies the reported *p*-values and 95% confidence intervals is likely violated; positive intraclass correlation among isolates from the same woman would tend to narrow the confidence intervals and inflate the apparent statistical significance, so all associations should be interpreted as isolate-level and hypothesis-generating rather than as precise patient-level estimates. A further limitation concerns the composition of the vaginal discharge group, which comprised cultures from both pregnant and non-pregnant women across the full age range, including peri- and post-menopausal patients, and therefore represents a mixed gynecological and obstetric population rather than a strictly perinatal one. Obstetric and fetal variables are undefined for non-pregnant women; accordingly, these variables were analyzed only within the pregnant stratum, and associations involving them should not be generalized to the vaginal discharge cohort as a whole. Finally, because the total number of cultures performed was not available, culture positivity, prevalence, incidence, and hospital-level burden could not be estimated; the reported proportions describe the relative species distribution among *Candida*-positive cultures and should not be interpreted as measures of frequency in the source obstetric and gynecological population. Furthermore, the analysed dataset comprised *Candida*-positive cultures identified through routine laboratory records, without accompanying symptom, clinical-diagnosis, microscopy, inflammatory, or treatment data; consequently, the retrospective design did not allow colonization to be consistently distinguished from clinically confirmed candidiasis, and the present results should be interpreted as reflecting culture positivity rather than documented *Candida* infection.

The clinical indication for each culture (for example, symptomatic infection versus routine screening) and data on recent antibiotic or antifungal exposure, hospitalization, cervical cerclage, and immunosuppression were not available from the laboratory records; these unrecorded factors may influence species distribution and contribute to selection bias. Several additional limitations should also be acknowledged. First, this was a retrospective, single-center study conducted at one tertiary obstetrics and gynecology hospital; the observed species distribution and associations may therefore not be generalizable to other populations, regions, or care settings with different case-mix, referral patterns, and laboratory practices. Second, because the data were extracted from routine laboratory and clinical records, several maternal, obstetric, and clinical variables were incompletely documented; missing values were handled by analyzing only the cultures for which the relevant variable was available, which reduced the effective sample size for some comparisons and may have introduced selection effects. Third, several comorbidities and clinical categories were represented by very few isolates, so the corresponding subgroup proportions and regression estimates are statistically unstable and should be regarded as hypothesis-generating; to mitigate this, continuous predictors were preferred and sparse categories were collapsed where feasible in the multivariable models. Fourth, a large number of variables were tested across the three sample types, so the univariate associations were exploratory in nature; although Benjamini–Hochberg correction was applied within each sample type to limit false-positive results, residual type I error cannot be excluded, and the associations require confirmation in independent cohorts. Species identification relied on chromogenic media with confirmatory VITEK^®^ testing; molecular or proteomic methods (MALDI-TOF MS or ITS sequencing) were not routinely available, which constrains the identification of uncommon species. Finally, antifungal susceptibility testing was not performed, so the clinical implications of the non-*albicans Candida* and *Pichia kudriavzevii* proportions—particularly regarding azole resistance—could not be evaluated and remain inferences drawn from the literature rather than from the present isolates.

Although several predictors were statistically significant, all models showed limited discrimination (AUC 0.60–0.68), low explained variance (Nagelkerke R^2^ 0.038–0.099), and poor sensitivity in most strata. The reversed sensitivity–specificity pattern in the non-pregnant stratum likely reflected class imbalance at the 0.5 probability threshold rather than strong predictive performance. Accordingly, these models should be interpreted as exploratory and hypothesis-generating rather than as clinically predictive tools. Taken together, these limitations indicate that the findings describe isolate-level patterns among clinician-selected *Candida*-positive cultures rather than infection prevalence, causal relationships, or clinically confirmed candidiasis in the source population. The aggregated non-*albicans Candida* outcome—including named species, *Candida* spp. isolates without definitive species-level identification, and six mixed cultures—was used only for exploratory modelling and should not be interpreted as species-specific. Because identification relied on definitive VITEK^®^ results without retained confidence or discordance data and without orthogonal molecular confirmation, uncommon species distributions require independent validation. The limited discrimination and explained variance of the models further support their interpretation as hypothesis-generating rather than clinically predictive.

## 5. Conclusions

*Candida albicans* was the predominant species across all sample types, while non-*albicans Candida*, particularly *Pichia kudriavzevii*, represented a substantial minority, consistent with the broader trend toward non-*albicans* species reported in the literature. Species distribution was compartment-dependent. In amniotic fluid, species distribution was largely independent of clinical variables; older maternal age and lower parity were the independent correlates of non-*albicans Candida*, whereas gestational age was not retained in multivariable analysis. In lochial cultures, parity remained the only association after false-discovery-rate correction, whereas maternal age and parity were retained as modest independent correlates in multivariable analysis, indicating a relatively homogeneous postpartum profile. In contrast, vaginal discharge demonstrated multiple significant associations, including maternal age, reproductive status, pregnancy characteristics, and selected comorbidities, reflecting a more complex host–microbiota interaction. *C. albicans* predominated in women of reproductive age and during pregnancy, whereas non-*albicans* species were relatively more frequent in older and non-pregnant women, consistent with an estrogen-dependent gynecological pattern rather than an association with adverse pregnancy or fetal outcomes. Multivariable models showed only limited discriminatory performance across all groups, indicating that species distribution is multifactorial and not fully explained by clinical variables. These findings emphasize the importance of species-level identification, particularly for non-*albicans Candida*, for microbiological surveillance and characterization of the species distribution among culture-positive isolates; assessing its implications for clinical management and antifungal stewardship would require antifungal susceptibility and treatment data, which were not available in this study. More broadly, by characterizing *Candida*-positive maternal and perinatal compartments that may contribute to fetal or neonatal microbial exposure, the study provides perinatal microbiological context that may support future integrated, complex microbiological evaluation of newborns; it does not, however, itself assess neonatal colonization, neonatal infection, or vertical transmission.

## Figures and Tables

**Table 1:** Association between *Candida albicans* and non-*albicans Candida* isolates and maternal, obstetric, and fetal characteristics in amniotic fluid cultures (N = 242).

Variable	Category	Total, n (%)	*Candida albicans*, n (%)	Non-*albicans Candida*, n (%)	*p*-Value
Maternal demographics					
Maternal age group	13–17 years	16 (6.6%)	14 (8.3%)	2 (2.7%)	0.133
	18–22 years	55 (22.7%)	45 (26.6%)	10 (13.7%)	
	23–27 years	65 (26.9%)	40 (23.7%)	25 (34.2%)	
	28–32 years	42 (17.4%)	27 (16.0%)	15 (20.5%)	
	33–37 years	48 (19.8%)	31 (18.3%)	17 (23.3%)	
	38–42 years	13 (5.4%)	10 (5.9%)	3 (4.1%)	
	43–47 years	3 (1.2%)	2 (1.2%)	1 (1.4%)	
Place of residence	Rural	169 (69.8%)	114 (67.5%)	55 (75.3%)	0.220
	Urban	73 (30.2%)	55 (32.5%)	18 (24.7%)	
Month of sampling	January	21 (8.7%)	13 (7.7%)	8 (11.0%)	**0.040**
	February	13 (5.4%)	7 (4.1%)	6 (8.2%)	
	March	23 (9.5%)	22 (13.0%)	1 (1.4%)	
	April	13 (5.4%)	12 (7.1%)	1 (1.4%)	
	May	24 (9.9%)	18 (10.7%)	6 (8.2%)	
	June	12 (5.0%)	9 (5.3%)	3 (4.1%)	
	July	21 (8.7%)	15 (8.9%)	6 (8.2%)	
	August	14 (5.8%)	6 (3.6%)	8 (11.0%)	
	September	24 (9.9%)	18 (10.7%)	6 (8.2%)	
	October	19 (7.9%)	12 (7.1%)	7 (9.6%)	
	November	35 (14.5%)	22 (13.0%)	13 (17.8%)	
	December	23 (9.5%)	15 (8.9%)	8 (11.0%)	
Maternal BMI	Normal weight	237 (97.9%)	168 (99.4%)	69 (94.5%)	**0.029**
	Obesity class I	4 (1.7%)	1 (0.6%)	3 (4.1%)	
	Obesity class II	1 (0.4%)	0 (0.0%)	1 (1.4%)	
Chronic smoking	Non-smoker	241 (99.6%)	168 (99.4%)	73 (100.0%)	1.000
	Smoker	1 (0.4%)	1 (0.6%)	0 (0.0%)	
Obstetric history and reproductive context					
Gravidity group	Primigravida	92 (38.0%)	62 (36.7%)	30 (41.1%)	0.138
	Multigravida	126 (52.1%)	86 (50.9%)	40 (54.8%)	
	Grand multigravida	24 (9.9%)	21 (12.4%)	3 (4.1%)	
Parity group	Nulliparous	10 (4.1%)	7 (4.1%)	3 (4.1%)	0.096
	Primiparous	111 (45.9%)	72 (42.6%)	39 (53.4%)	
	Multiparous	110 (45.5%)	79 (46.7%)	31 (42.5%)	
	Grand multiparous	11 (4.5%)	11 (6.5%)	0 (0.0%)	
Previous caesarean section	Absent	214 (88.4%)	150 (88.8%)	64 (87.7%)	0.808
	Present	28 (11.6%)	19 (11.2%)	9 (12.3%)	
In vitro fertilization (IVF)	Absent	236 (97.5%)	165 (97.6%)	71 (97.3%)	1.000
	Present	6 (2.5%)	4 (2.4%)	2 (2.7%)	
Current pregnancy and intrapartum features					
Gestational age group	First trimester	2 (0.8%)	0 (0.0%)	2 (2.7%)	**0.001**
	Second trimester	17 (7.0%)	7 (4.1%)	10 (13.7%)	
	Third trimester	223 (92.1%)	162 (95.9%)	61 (83.6%)	
Status of fetal membranes	Spontaneously ruptured	187 (77.3%)	133 (78.7%)	54 (74.0%)	0.778
	Intact	52 (21.5%)	34 (20.1%)	18 (24.7%)	
	Fissured	3 (1.2%)	2 (1.2%)	1 (1.4%)	
Onset of labor	No labor	101 (41.7%)	65 (38.5%)	36 (49.3%)	0.272
	Pre-labor	80 (33.1%)	60 (35.5%)	20 (27.4%)	
	Induced	61 (25.2%)	44 (26.0%)	17 (23.3%)	
Pregnancy complication (antepartum)	None	165 (68.2%)	119 (70.4%)	46 (63.0%)	0.129
	Threatened preterm labor	52 (21.5%)	38 (22.5%)	14 (19.2%)	
	Threatened miscarriage	12 (5.0%)	4 (2.4%)	8 (11.0%)	
	IUGR/Threatened preterm labor	2 (0.8%)	1 (0.6%)	1 (1.4%)	
	IUGR	3 (1.2%)	2 (1.2%)	1 (1.4%)	
	Polyhydramnios/IUGR	1 (0.4%)	1 (0.6%)	0 (0.0%)	
	Polyhydramnios	2 (0.8%)	1 (0.6%)	1 (1.4%)	
	Polyhydramnios/Threatened preterm labor	2 (0.8%)	1 (0.6%)	1 (1.4%)	
	Placenta praevia	1 (0.4%)	0 (0.0%)	1 (1.4%)	
	Placenta praevia/Threatened preterm labor	2 (0.8%)	2 (1.2%)	0 (0.0%)	
Vaginal bleeding	Absent	241 (99.6%)	169 (100.0%)	72 (98.6%)	0.302
	Present	1 (0.4%)	0 (0.0%)	1 (1.4%)	
Vaginal infections	Absent	239 (98.8%)	168 (99.4%)	71 (97.3%)	0.217
	Present	3 (1.2%)	1 (0.6%)	2 (2.7%)	
Obstetric hemorrhage	Absent	241 (99.6%)	169 (100.0%)	72 (98.6%)	0.302
	Present	1 (0.4%)	0 (0.0%)	1 (1.4%)	
Rh incompatibility	Absent	218 (90.1%)	156 (92.3%)	62 (84.9%)	0.078
	Present	24 (9.9%)	13 (7.7%)	11 (15.1%)	
Maternal comorbidities					
Diabetes	No diabetes	238 (98.3%)	167 (98.8%)	71 (97.3%)	0.454
	Gestational diabetes mellitus	3 (1.2%)	1 (0.6%)	2 (2.7%)	
	Type 1 diabetes mellitus	1 (0.4%)	1 (0.6%)	0 (0.0%)	
Pregnancy-induced hypertension	Absent	231 (95.5%)	161 (95.3%)	70 (95.9%)	1.000
	Present	11 (4.5%)	8 (4.7%)	3 (4.1%)	
Thyroid disorders	Absent	240 (99.2%)	167 (98.8%)	73 (100.0%)	1.000
	Present	2 (0.8%)	2 (1.2%)	0 (0.0%)	
Inherited thrombophilia	Absent	230 (95.0%)	164 (97.0%)	66 (90.4%)	**0.048**
	Present	12 (5.0%)	5 (3.0%)	7 (9.6%)	
History of renal disease	Absent	239 (98.8%)	166 (98.2%)	73 (100.0%)	0.556
	Present	3 (1.2%)	3 (1.8%)	0 (0.0%)	
Hepatitis	Absent	241 (99.6%)	169 (100.0%)	72 (98.6%)	0.302
	Hepatitis B	1 (0.4%)	0 (0.0%)	1 (1.4%)	
Ocular disorders	Absent	239 (98.8%)	168 (99.4%)	71 (97.3%)	0.217
	Present	3 (1.2%)	1 (0.6%)	2 (2.7%)	
Uterine leiomyoma	Absent	237 (97.9%)	167 (98.8%)	70 (95.9%)	0.163
	Present	5 (2.1%)	2 (1.2%)	3 (4.1%)	
Fetal characteristics and outcomes					
Fetal presentation at delivery	Cephalic	220 (90.9%)	154 (91.1%)	66 (90.4%)	0.759
	Breech	18 (7.4%)	12 (7.1%)	6 (8.2%)	
	Transverse lie	2 (0.8%)	2 (1.2%)	0 (0.0%)	
	Cephalic/Transverse lie (twin pregnancy)	1 (0.4%)	1 (0.6%)	0 (0.0%)	
	Not recorded	1 (0.4%)	0 (0.0%)	1 (1.4%)	
Fetal conditions	None	207 (85.5%)	149 (88.2%)	58 (79.5%)	0.187
	Single nuchal cord	27 (11.2%)	16 (9.5%)	11 (15.1%)	
	Double nuchal cord	6 (2.5%)	3 (1.8%)	3 (4.1%)	
	Abnormal fetal Doppler findings	1 (0.4%)	1 (0.6%)	0 (0.0%)	
	Fetal duodenal stenosis	1 (0.4%)	0 (0.0%)	1 (1.4%)	
Fetal outcome	Live singleton fetus	241 (99.6%)	168 (99.4%)	73 (100.0%)	1.000
	Live twin fetuses	1 (0.4%)	1 (0.6%)	0 (0.0%)	

**Notes:** Data are presented as n (%). Percentages in the Total column are calculated relative to the applicable denominator (N = 242 for all variables in this cohort). Percentages for *Candida albicans* and non-*albicans Candida* are within-group (column) percentages, calculated relative to the respective species-group totals (*Candida albicans*, n = 169; non-*albicans Candida*, n = 73). The single co-isolation of *C. albicans* and *Nakaseomyces glabratus* was assigned to the non-*albicans Candida* group. **Statistical tests:** Between-group comparisons used Pearson’s chi-square test when all expected cell counts were ≥5, Fisher’s exact test for 2 × 2 tables with any expected count < 5, and a Monte Carlo approximation of the exact test (chi-square statistic; 100,000 resamples) for larger tables with any expected count < 5. Reported *p*-values are two-sided and unadjusted; values in bold denote *p* < 0.05. False-discovery-rate correction was applied separately within each sample type (amniotic fluid, lochia, and vaginal discharge) using the Benjamini–Hochberg procedure; within the amniotic fluid cohort, only gestational age group remained significant after correction (q = 0.028), and all other associations had q ≥ 0.34. Parity and gravidity: Parity was categorized as nulliparous (0 births), primiparous (1 birth), multiparous (2–4 births), and grand multiparous (≥5 births); gravidity as primigravida (1 pregnancy), multigravida (2–4 pregnancies), and grand multigravida (≥5 pregnancies). The same thresholds are applied throughout. Fetal presentation: One record contained “cervical insufficiency” in the fetal-presentation field and was treated as missing/not recorded; the association test for this variable was therefore based on 241 records. Abbreviations: BMI, body mass index; IUGR, intrauterine growth restriction; IVF, in vitro fertilization; Rh, rhesus factor.

**Table 2:** Association between *Candida* species distribution and maternal, obstetric, and fetal characteristics in lochia cultures.

Variable	Category	Total (N = 827), n (%)	*Candida albicans* (n = 553), n (%)	Non-*albicans Candida* (n = 274), n (%)	*p*-Value
Maternal demographics					
Month of sampling	January	68 (8.2%)	41 (7.4%)	27 (9.9%)	0.110
	February	83 (10.0%)	49 (8.9%)	34 (12.4%)	
	March	73 (8.8%)	56 (10.1%)	17 (6.2%)	
	April	54 (6.5%)	35 (6.3%)	19 (6.9%)	
	May	68 (8.2%)	38 (6.9%)	30 (10.9%)	
	June	48 (5.8%)	29 (5.2%)	19 (6.9%)	
	July	68 (8.2%)	47 (8.5%)	21 (7.7%)	
	August	63 (7.6%)	47 (8.5%)	16 (5.8%)	
	September	78 (9.4%)	56 (10.1%)	22 (8.0%)	
	October	91 (11.0%)	66 (11.9%)	25 (9.1%)	
	November	69 (8.3%)	43 (7.8%)	26 (9.5%)	
	December	64 (7.7%)	46 (8.3%)	18 (6.6%)	
Maternal age group	13–17 years	37 (4.5%)	31 (5.6%)	6 (2.2%)	**0.009**
	18–22 years	108 (13.1%)	84 (15.2%)	24 (8.8%)	
	23–27 years	203 (24.5%)	132 (23.9%)	71 (25.9%)	
	28–32 years	230 (27.8%)	153 (27.7%)	77 (28.1%)	
	33–37 years	181 (21.9%)	108 (19.5%)	73 (26.6%)	
	38–42 years	61 (7.4%)	41 (7.4%)	20 (7.3%)	
	43–47 years	6 (0.7%)	4 (0.7%)	2 (0.7%)	
	48–52 years	1 (0.1%)	0 (0.0%)	1 (0.4%)	
Place of residence	Rural	490 (59.3%)	342 (61.8%)	148 (54.0%)	**0.035**
	Urban	337 (40.7%)	211 (38.2%)	126 (46.0%)	
Maternal BMI	Normal weight	810 (97.9%)	540 (97.6%)	270 (98.5%)	**0.044**
	Obesity class I	15 (1.8%)	13 (2.4%)	2 (0.7%)	
	Obesity class II	1 (0.1%)	0 (0.0%)	1 (0.4%)	
	Obesity class III	1 (0.1%)	0 (0.0%)	1 (0.4%)	
Chronic smoking	Non-smoker	822 (99.4%)	548 (99.1%)	274 (100.0%)	0.177
	Smoker	5 (0.6%)	5 (0.9%)	0 (0.0%)	
Obstetric history and reproductive context					
Gravidity group	Primigravida	303 (36.6%)	189 (34.2%)	114 (41.6%)	0.068
	Multigravida	460 (55.6%)	316 (57.1%)	144 (52.6%)	
	Grand multigravida	64 (7.7%)	48 (8.7%)	16 (5.8%)	
Parity group	Nulliparous	9 (1.1%)	3 (0.5%)	6 (2.2%)	**<0.001**
	Primiparous	371 (44.9%)	230 (41.6%)	141 (51.5%)	
	Multiparous	422 (51.0%)	298 (53.9%)	124 (45.3%)	
	Grand multiparous	25 (3.0%)	22 (4.0%)	3 (1.1%)	
Previous caesarean section	Absent	603 (72.9%)	400 (72.3%)	203 (74.1%)	0.619
	Present	224 (27.1%)	153 (27.7%)	71 (25.9%)	
In vitro fertilization (IVF)	Absent	790 (95.5%)	529 (95.7%)	261 (95.3%)	0.858
	Present	37 (4.5%)	24 (4.3%)	13 (4.7%)	
Current pregnancy and intrapartum features					
Gestational age group	Second trimester	4 (0.5%)	1 (0.2%)	3 (1.1%)	0.109
	Third trimester	823 (99.5%)	552 (99.8%)	271 (98.9%)	
Status of fetal membranes	Spontaneously ruptured	96 (11.6%)	66 (11.9%)	30 (10.9%)	0.756
	Intact	729 (88.1%)	486 (87.9%)	243 (88.7%)	
	Fissured	2 (0.2%)	1 (0.2%)	1 (0.4%)	
Onset of labor	Pre-labor	643 (77.8%)	428 (77.4%)	215 (78.5%)	0.790
	Induced	184 (22.2%)	125 (22.6%)	59 (21.5%)	
Pregnancy complication (antepartum)	None	645 (78.0%)	443 (80.1%)	202 (73.7%)	0.122
	Placenta praevia	94 (11.4%)	55 (9.9%)	39 (14.2%)	
	Threatened preterm labor	39 (4.7%)	27 (4.9%)	12 (4.4%)	
	IUGR	23 (2.8%)	16 (2.9%)	7 (2.6%)	
	Oligohydramnios	9 (1.1%)	5 (0.9%)	4 (1.5%)	
	Preeclampsia	3 (0.4%)	2 (0.4%)	1 (0.4%)	
	Polyhydramnios	2 (0.2%)	1 (0.2%)	1 (0.4%)	
	Chorioamnionitis	1 (0.1%)	0 (0.0%)	1 (0.4%)	
	Multiple/combined complications	11 (1.3%)	4 (0.7%)	7 (2.6%)	
Vaginal bleeding	Absent	817 (98.8%)	546 (98.7%)	271 (98.9%)	1.000
	Present	10 (1.2%)	7 (1.3%)	3 (1.1%)	
Vaginal infections	Absent	814 (98.4%)	546 (98.7%)	268 (97.8%)	0.375
	Present	13 (1.6%)	7 (1.3%)	6 (2.2%)	
Obstetric hemorrhage	Absent	817 (98.8%)	547 (98.9%)	270 (98.5%)	0.738
	Present	10 (1.2%)	6 (1.1%)	4 (1.5%)	
Rh incompatibility	Absent	790 (95.5%)	526 (95.1%)	264 (96.4%)	0.479
	Present	37 (4.5%)	27 (4.9%)	10 (3.6%)	
Maternal comorbidities					
Diabetes	No diabetes	793 (95.9%)	531 (96.0%)	262 (95.6%)	0.819
	Gestational diabetes mellitus	29 (3.5%)	18 (3.3%)	11 (4.0%)	
	Type 1 diabetes mellitus	2 (0.2%)	2 (0.4%)	0 (0.0%)	
	Type 2 diabetes mellitus	3 (0.4%)	2 (0.4%)	1 (0.4%)	
Pregnancy-induced hypertension	Absent	783 (94.7%)	523 (94.6%)	260 (94.9%)	1.000
	Present	44 (5.3%)	30 (5.4%)	14 (5.1%)	
Hypothyroidism	Absent	818 (98.9%)	547 (98.9%)	271 (98.9%)	1.000
	Present	9 (1.1%)	6 (1.1%)	3 (1.1%)	
Inherited thrombophilia	Absent	772 (93.3%)	521 (94.2%)	251 (91.6%)	0.182
	Present	55 (6.7%)	32 (5.8%)	23 (8.4%)	
History of renal disease	Absent	817 (98.8%)	549 (99.3%)	268 (97.8%)	0.091
	Present	10 (1.2%)	4 (0.7%)	6 (2.2%)	
**Hepatitis**	Absent	821 (99.3%)	549 (99.3%)	272 (99.3%)	0.777
	Hepatitis B	3 (0.4%)	2 (0.4%)	1 (0.4%)	
	Hepatitis C	1 (0.1%)	1 (0.2%)	0 (0.0%)	
	Hepatitis B/Hepatitis C co-infection	1 (0.1%)	1 (0.2%)	0 (0.0%)	
	Hepatic abscess	1 (0.1%)	0 (0.0%)	1 (0.4%)	
**Ocular disorders**	Absent	810 (97.9%)	544 (98.4%)	266 (97.1%)	0.296
	Present	17 (2.1%)	9 (1.6%)	8 (2.9%)	
**Uterine leiomyoma**	Absent	817 (98.8%)	546 (98.7%)	271 (98.9%)	1.000
	Present	10 (1.2%)	7 (1.3%)	3 (1.1%)	
**Intervertebral disc herniation**	Absent	823 (99.5%)	552 (99.8%)	271 (98.9%)	0.109
	Present	4 (0.5%)	1 (0.2%)	3 (1.1%)	
**Autoimmune thyroiditis**	Absent	818 (98.9%)	549 (99.3%)	269 (98.2%)	0.167
	Present	9 (1.1%)	4 (0.7%)	5 (1.8%)	
**Cholelithiasis**	Absent	826 (99.9%)	553 (100.0%)	273 (99.6%)	0.331
	Present	1 (0.1%)	0 (0.0%)	1 (0.4%)	
**Sinus tachycardia**	Absent	825 (99.8%)	553 (100.0%)	272 (99.3%)	0.110
	Present	2 (0.2%)	0 (0.0%)	2 (0.7%)	
**Bronchial asthma**	Absent	824 (99.6%)	552 (99.8%)	272 (99.3%)	0.256
	Present	3 (0.4%)	1 (0.2%)	2 (0.7%)	
**Gestational edema**	Absent	822 (99.4%)	550 (99.5%)	272 (99.3%)	0.668
	Present	5 (0.6%)	3 (0.5%)	2 (0.7%)	
***Fetal characteristics and outcomes***					
**Fetal presentation at delivery**	Cephalic (singleton)	779 (94.2%)	520 (94.0%)	259 (94.5%)	0.624
	Breech (singleton)	28 (3.4%)	21 (3.8%)	7 (2.6%)	
	Transverse lie (singleton)	6 (0.7%)	3 (0.5%)	3 (1.1%)	
	Twin/multiple pregnancy	14 (1.7%)	9 (1.6%)	5 (1.8%)	
**Fetal conditions**	None	706 (85.4%)	475 (85.9%)	231 (84.3%)	0.525
	Single nuchal cord	100 (12.1%)	67 (12.1%)	33 (12.0%)	
	Double nuchal cord	10 (1.2%)	6 (1.1%)	4 (1.5%)	
	Abnormal fetal Doppler findings	4 (0.5%)	2 (0.4%)	2 (0.7%)	
	Fetal arrhythmia	1 (0.1%)	0 (0.0%)	1 (0.4%)	
	Fetal macrosomia	5 (0.6%)	2 (0.4%)	3 (1.1%)	
	Fetal hydronephrosis with ureteral dilatation	1 (0.1%)	1 (0.2%)	0 (0.0%)	
**Fetal outcome**	Live singleton fetus	812 (98.2%)	543 (98.2%)	269 (98.2%)	0.853
	Live twin fetuses	14 (1.7%)	9 (1.6%)	5 (1.8%)	
	Non-viable fetus (intrauterine fetal demise)	1 (0.1%)	1 (0.2%)	0 (0.0%)	

**Notes:** BMI = body mass index; IUGR = intrauterine growth restriction. All data are presented as n (%). Percentages in the “Total” column are calculated relative to all cases (N = 827), whereas percentages for *Candida albicans* and non-*albicans Candida* represent within-group (column) percentages relative to the respective species-group totals (n = 553 and n = 274). Categorical associations were tested using the chi-square test when all expected cell counts were ≥5, Fisher’s exact test for 2 × 2 tables, and a Monte Carlo approximation of the exact (Fisher–Freeman–Halton) test with 10,000 resamples for larger tables with any expected count < 5. Reported *p*-values are unadjusted; false-discovery-rate control (Benjamini–Hochberg, applied within each sample type; Section 2.4) and its interpretation are summarized in Section 3 and Section 4. Statistically significant associations (*p* < 0.05) are shown in bold.

**Table 3:** Association between *Candida* species distribution and maternal, obstetric, and fetal characteristics in vaginal discharge.

Variable	Category	Total *n* (%)	*Candida albicans*, *n* (%)	Non-*albicans Candida*, *n* (%)	*p*-Value
Maternal demographics					
Month of sampling	January	160 (7.9%)	83 (7.4%)	77 (8.5%)	**<0.001**
	February	158 (7.8%)	88 (7.9%)	70 (7.8%)	
	March	129 (6.4%)	70 (6.3%)	59 (6.5%)	
	April	113 (5.6%)	74 (6.6%)	39 (4.3%)	
	May	147 (7.3%)	81 (7.2%)	66 (7.3%)	
	June	135 (6.7%)	66 (5.9%)	69 (7.6%)	
	July	167 (8.3%)	99 (8.9%)	68 (7.5%)	
	August	185 (9.2%)	104 (9.3%)	81 (9.0%)	
	September	202 (10.0%)	127 (11.4%)	75 (8.3%)	
	October	242 (12.0%)	129 (11.5%)	113 (12.5%)	
	November	208 (10.3%)	86 (7.7%)	122 (13.5%)	
	December	174 (8.6%)	111 (9.9%)	63 (7.0%)	
Age group	≤17 years	70 (3.5%)	57 (5.1%)	13 (1.4%)	**<0.001**
	18–22 years	254 (12.6%)	178 (15.9%)	76 (8.4%)	
	23–27 years	495 (24.5%)	309 (27.6%)	186 (20.6%)	
	28–32 years	432 (21.4%)	246 (22.0%)	186 (20.6%)	
	33–37 years	395 (19.6%)	206 (18.4%)	189 (21.0%)	
	38–42 years	148 (7.3%)	70 (6.3%)	78 (8.6%)	
	43–47 years	71 (3.5%)	27 (2.4%)	44 (4.9%)	
	48–52 years	43 (2.1%)	6 (0.5%)	37 (4.1%)	
	53–57 years	29 (1.4%)	3 (0.3%)	26 (2.9%)	
	58–62 years	20 (1.0%)	3 (0.3%)	17 (1.9%)	
	63–67 years	27 (1.3%)	4 (0.4%)	23 (2.5%)	
	68–72 years	15 (0.7%)	2 (0.2%)	13 (1.4%)	
	73–77 years	15 (0.7%)	4 (0.4%)	11 (1.2%)	
	78–82 years	5 (0.2%)	3 (0.3%)	2 (0.2%)	
	83–87 years	1 (0.0%)	0 (0.0%)	1 (0.1%)	
Place of residence	Rural	1247 (61.7%)	705 (63.1%)	542 (60.1%)	0.172
	Urban	773 (38.3%)	413 (36.9%)	360 (39.9%)	
Maternal BMI	Normal weight	1989 (98.5%)	1097 (98.1%)	892 (98.9%)	0.456
	Obesity class I	27 (1.3%)	18 (1.6%)	9 (1.0%)	
	Obesity class II	2 (0.1%)	1 (0.1%)	1 (0.1%)	
	Obesity class III	2 (0.1%)	2 (0.2%)	0 (0.0%)	
Chronic smoking	Non-smoker	2008 (99.4%)	1108 (99.1%)	900 (99.8%)	0.050
	Smoker	12 (0.6%)	10 (0.9%)	2 (0.2%)	
Obstetric history and reproductive context					
Gravidity group	Nulligravida	622 (30.8%)	261 (23.3%)	361 (40.0%)	**<0.001**
	Primigravida	514 (25.4%)	329 (29.4%)	185 (20.5%)	
	Multigravida	665 (32.9%)	385 (34.4%)	280 (31.0%)	
	Grand multigravida	219 (10.8%)	143 (12.8%)	76 (8.4%)	
Parity group	Nulliparous	839 (41.5%)	378 (33.8%)	461 (51.1%)	**<0.001**
	Primiparous	549 (27.2%)	356 (31.8%)	193 (21.4%)	
	Multiparous	594 (29.4%)	355 (31.8%)	239 (26.5%)	
	Grand multiparous	38 (1.9%)	29 (2.6%)	9 (1.0%)	
Previous caesarean section	Absent	1728 (85.5%)	937 (83.8%)	791 (87.7%)	**0.014**
	Present	292 (14.5%)	181 (16.2%)	111 (12.3%)	
In vitro fertilization (IVF)	Absent	1988 (98.4%)	1101 (98.5%)	887 (98.3%)	0.799
	Present	32 (1.6%)	17 (1.5%)	15 (1.7%)	
Infertility	Without infertility	1998 (98.9%)	1113 (99.6%)	885 (98.1%)	**0.002**
	Primary infertility	8 (0.4%)	3 (0.3%)	5 (0.6%)	
	Secondary infertility	14 (0.7%)	2 (0.2%)	12 (1.3%)	
Current pregnancy and intrapartum features					
Gestational age group	First trimester	237 (14.6%)	124 (12.4%)	113 (18.0%)	**0.003**
	Second trimester	355 (21.8%)	212 (21.2%)	143 (22.7%)	
	Third trimester	1035 (63.6%)	662 (66.3%)	373 (59.3%)	
Status of fetal membranes	Spontaneously ruptured	13 (1.4%)	9 (1.5%)	4 (1.1%)	0.858
	Intact	943 (98.5%)	597 (98.4%)	346 (98.9%)	
	Fissured	1 (0.1%)	1 (0.2%)	0 (0.0%)	
Onset of labor	No labor	1221 (75.0%)	725 (72.6%)	496 (78.9%)	**0.005**
	Pre-labor	406 (25.0%)	273 (27.4%)	133 (21.1%)	
Pregnancy complication (antepartum)	None	1204 (74.0%)	747 (74.8%)	457 (72.7%)	0.129
	Threatened preterm labor	218 (13.4%)	142 (14.2%)	76 (12.1%)	
	Threatened miscarriage	98 (6.0%)	50 (5.0%)	48 (7.6%)	
	Placenta praevia	53 (3.3%)	27 (2.7%)	26 (4.1%)	
	IUGR	12 (0.7%)	9 (0.9%)	3 (0.5%)	
	Oligohydramnios	10 (0.6%)	7 (0.7%)	3 (0.5%)	
	Preeclampsia	3 (0.2%)	2 (0.2%)	1 (0.2%)	
	Polyhydramnios	3 (0.2%)	2 (0.2%)	1 (0.2%)	
	Multiple/combined complications	26 (1.6%)	12 (1.2%)	14 (2.2%)	
Vaginal bleeding	Absent	2012 (99.6%)	1113 (99.6%)	899 (99.7%)	0.738
	Present	8 (0.4%)	5 (0.4%)	3 (0.3%)	
Vaginal infections	Absent	2005 (99.3%)	1110 (99.3%)	895 (99.2%)	0.875
	Present	15 (0.7%)	8 (0.7%)	7 (0.8%)	
Obstetric hemorrhage	Absent	1957 (96.9%)	1093 (97.8%)	864 (95.8%)	**0.011**
	Present	63 (3.1%)	25 (2.2%)	38 (4.2%)	
Rh incompatibility	Absent	1980 (98.0%)	1096 (98.0%)	884 (98.0%)	0.964
	Present	40 (2.0%)	22 (2.0%)	18 (2.0%)	
Maternal comorbidities					
Diabetes	No diabetes	1967 (97.4%)	1090 (97.5%)	877 (97.2%)	**0.014**
	Gestational diabetes mellitus	30 (1.5%)	21 (1.9%)	9 (1.0%)	
	Type 1 diabetes mellitus	8 (0.4%)	4 (0.4%)	4 (0.4%)	
	Type 2 diabetes mellitus	15 (0.7%)	3 (0.3%)	12 (1.3%)	
Pregnancy-induced hypertension/HTA	Absent	1923 (95.2%)	1072 (95.9%)	851 (94.3%)	0.108
	Present	97 (4.8%)	46 (4.1%)	51 (5.7%)	
Hypothyroidism	Absent	1994 (98.7%)	1110 (99.3%)	884 (98.0%)	**0.011**
	Present	26 (1.3%)	8 (0.7%)	18 (2.0%)	
Inherited thrombophilia/Thrombocytopenia	Absent	1934 (95.7%)	1072 (95.9%)	862 (95.6%)	0.723
	Present	86 (4.3%)	46 (4.1%)	40 (4.4%)	
History of renal disease	Absent	1973 (97.7%)	1097 (98.1%)	876 (97.1%)	0.137
	Present	47 (2.3%)	21 (1.9%)	26 (2.9%)	
Hepatitis/HIV	Absent	2009 (99.5%)	1113 (99.6%)	896 (99.3%)	0.746
	Hepatitis B	8 (0.4%)	3 (0.3%)	5 (0.6%)	
	Hepatitis C	2 (0.1%)	1 (0.1%)	1 (0.1%)	
	HIV	1 (0.0%)	1 (0.1%)	0 (0.0%)	
Ocular disorders	Absent	2004 (99.2%)	1108 (99.1%)	896 (99.3%)	0.563
	Present	16 (0.8%)	10 (0.9%)	6 (0.7%)	
Uterine leiomyoma	Absent	1926 (95.3%)	1093 (97.8%)	833 (92.4%)	**<0.001**
	Present	94 (4.7%)	25 (2.2%)	69 (7.6%)	
History of neoplasia	Absent	1991 (98.6%)	1107 (99.0%)	884 (98.0%)	0.057
	Present	29 (1.4%)	11 (1.0%)	18 (2.0%)	
Fetal characteristics and outcomes					
Fetal presentation at delivery	Cephalic	863 (91.5%)	556 (93.1%)	307 (88.7%)	0.059
	Breech	62 (6.6%)	31 (5.2%)	31 (9.0%)	
	Transverse lie	18 (1.9%)	10 (1.7%)	8 (2.3%)	
Fetal conditions	None	1492 (91.7%)	915 (91.7%)	577 (91.7%)	0.729
	Single nuchal cord	101 (6.2%)	64 (6.4%)	37 (5.9%)	
	Double nuchal cord	1 (0.1%)	0 (0.0%)	1 (0.2%)	
	Abnormal fetal Doppler findings	5 (0.3%)	2 (0.2%)	3 (0.5%)	
	Intrauterine fetal demise	16 (1.0%)	9 (0.9%)	7 (1.1%)	
	Other rare conditions	12 (0.7%)	8 (0.8%)	4 (0.6%)	
Fetal outcome	Live singleton fetus	1580 (97.1%)	974 (97.6%)	606 (96.3%)	0.297
	Live twin fetuses	31 (1.9%)	15 (1.5%)	16 (2.5%)	
	Non-viable fetus (intrauterine fetal demise)	16 (1.0%)	9 (0.9%)	7 (1.1%)	

Notes: BMI, body mass index; IUGR, intrauterine growth restriction; HTA, arterial hypertension; IVF, in vitro fertilization; HIV, human immunodeficiency virus. All data are presented as *n* (%). Percentages in the Total column are calculated relative to the applicable denominator for each variable; percentages for *Candida albicans* and non-*albicans Candida* are within-group percentages, calculated relative to the total number of isolates in each species group (*C. albicans*, n = 1118; non-*albicans Candida*, n = 902). For variables applicable to the whole cohort, the denominator is the complete set of vaginal discharge isolates (N = 2020). For pregnancy- and fetus-specific variables (gestational age, status of the fetal membranes, onset of labour, antepartum pregnancy complication, fetal presentation, fetal conditions, and fetal outcome), which apply only to women who were pregnant at the time of sampling, percentages are calculated among currently pregnant patients (valid n = 1627), and the 393 non-pregnant women were excluded as not applicable; for status of the fetal membranes and fetal presentation, percentages are further restricted to patients with a documented value (n = 957 and n = 943, respectively), with observations recorded as “not recorded” excluded as missing data. For antepartum pregnancy complications and fetal conditions, “None” denotes the absence of a documented finding (a true negative) rather than missing data. “Other rare conditions” includes fetal macrosomia, congenital malformations, triple nuchal cord, and other isolated cases. Categorical associations were assessed using Pearson’s chi-square test when all expected cell counts were ≥5; Fisher’s exact test for 2 × 2 tables with any expected count < 5; and a Monte Carlo exact test (10,000 resamples) for tables with three or more categories and any expected count < 5. Reported *p*-values are two-sided and unadjusted; false-discovery-rate control (Benjamini–Hochberg, applied within each sample type) is summarised separately (Section 2.4). Values *p* < 0.05 are shown in bold and were considered statistically significant.

**Table 4:** Multivariable logistic regression models for non-*albicans Candida* isolation, by sample type.

Predictor	Reference/Scale	OR	95% CI	*p*-Value
*Amniotic fluid (n = 242); outcome: non-albicans Candida (1)* vs. *C. albicans (0)*				
Maternal age	per 1-year increase	1.07	1.02–1.12	0.003
Parity	per 1-birth increase	0.68	0.52–0.90	0.007
Gestational age	per 1-week increase	0.96	0.91–1.01	0.121
*Model performance: Nagelkerke R^2^ = 0.099; classification accuracy = 71.9%; sensitivity (non-albicans Candida) = 12.3%; specificity (C. albicans) = 97.6%; AUC = 0.65*				
*Lochia (n = 827); outcome: non-albicans Candida (1)* vs. *C. albicans (0)*				
Maternal age	per 1-year increase	1.05	1.03–1.08	<0.001
Parity	per 1-birth increase	0.76	0.65–0.88	<0.001
*Model performance: Nagelkerke R^2^ = 0.045; classification accuracy = 65.8%; sensitivity (non-albicans Candida) = 3.3%; specificity (C. albicans) = 96.7%; AUC = 0.63*				
*Vaginal discharge, pregnant stratum (n = 1627); outcome: non-albicans Candida (1)* vs. *C. albicans (0)*				
Maternal age	per 1-year increase	1.05	1.03–1.06	<0.001
Thyroid disorder, present vs absent	Reference: absent	2.90	1.14–7.35	0.025
Gestational age	per 1-week increase	0.99	0.98–1.00	0.062
Parity	per 1-birth increase	0.93	0.85–1.01	0.082
*Model performance: Nagelkerke R^2^ = 0.038; classification accuracy = 61.3%; sensitivity (non-albicans Candida) = 11.6%; specificity (C. albicans) = 92.7%; AUC = 0.60*				
*Vaginal discharge, non-pregnant stratum (n = 393); outcome: non-albicans Candida (1)* vs. *C. albicans (0)*				
Maternal age	per 1-year increase	1.04	1.02–1.06	<0.001
Parity	per 1-birth increase	1.02	0.33–3.10	0.974
*Model performance: Nagelkerke R^2^ = 0.095; classification accuracy = 71.5%; sensitivity (non-albicans Candida) = 96.7%; specificity (C. albicans) = 14.2%; AUC = 0.68. In this stratum, maternal age was the only statistically significant independent predictor (parity was non-significant).*				

**Notes:** OR, odds ratio; CI, confidence interval; AUC, area under the receiver operating characteristic curve. All models used non-*albicans Candida* as the outcome category coded as 1, with *Candida albicans* coded as 0. Continuous predictors were entered per one-unit increase. Binary predictors are shown as present versus absent unless otherwise specified. Polymicrobial co-isolations of *C. albicans* with a non-*albicans* species were assigned to the non-*albicans Candida* group. Odds ratios are reported with Wald-type 95% confidence intervals; sensitivity denotes correct classification of non-*albicans Candida* and specificity correct classification of *C. albicans* at a 0.5 probability threshold. Accuracy, sensitivity, specificity, and AUC represent apparent, in-sample performance estimates and were not internally validated; they should therefore be interpreted as descriptive measures of exploratory models rather than as validated predictive performance. Maternal BMI and other variables with extremely sparse categories were not entered into the models. All four models were statistically significant overall (omnibus likelihood-ratio test, *p* < 0.001). Estimates were recomputed from the 2024 isolate-level database of *Candida*-positive cultures (242 amniotic fluid, 827 lochial, and 2020 vaginal discharge cultures). Species-level descriptive analysis of the non-*albicans* group showed that *Pichia kudriavzevii* was the predominant named species in all three sample types, accounting for 19.0% of all amniotic fluid isolates, 26.58% of vaginal discharge isolates, and 21.3% of lochial isolates. *Candida* spp. isolates without definitive species-level identification accounted for 8.7%, 14.0%, and 9.7%, respectively, whereas *Nakaseomyces glabratus* accounted for 2.5%, 4.06%, and 2.1%. *Candida parapsilosis* was detected only in vaginal discharge cultures (0.05%).

**Table 5:** Distribution of non-*albicans Candida* species across sample types, n (% of *Candida*-positive isolates within each compartment).

Species	Amniotic Fluid (n = 242)	Vaginal Discharge (n = 2020)	Lochia (n = 827)
*Pichia kudriavzevii*	46 (19.0)	537 (26.6)	177 (21.3)
*Nakaseomyces glabratus*	6 (2.5)	82 (4.1)	17 (2.1)
*Candida parapsilosis*	0 (—)	1 (0.05)	0 (—)
*Candida* spp. (genus-level)	21 (8.7)	282 (14.0)	80 (9.7)
Total non-*albicans Candida*	73 (30.2)	902 (44.7)	274 (33.1)

Notes: Data are n (%) of all *Candida*-positive isolates within each sample type; *Candida albicans* accounted for the remainder (69.8%, 55.3%, and 66.9%, respectively). *Pichia kudriavzevii* was the predominant non-*albicans* species in all three compartments. “*Candida* spp. (genus-level)” denotes isolates confirmed as *Candida* at the genus level without a species-level VITEK^®^ identification. Dash (—), not detected. Percentages are rounded to one decimal. Co-isolations (n = 6) are counted under their non-*albicans* component.

## Data Availability

The data presented in this study are available on request from the corresponding author. The data are not publicly available due to privacy and ethical restrictions.
